# External Validation of a 5-Factor Risk Model for Breast Cancer–Related Lymphedema

**DOI:** 10.1001/jamanetworkopen.2024.55383

**Published:** 2025-01-21

**Authors:** Cherie Lin, Jie Su, Alison J. Wu, Neil Lin, Madison-Shira Hossack, Wei Shi, Wei Xu, Fei-Fei Liu, Jennifer Y. Y. Kwan

**Affiliations:** 1 Institute of Medical Science, Temerty Faculty of Medicine, University of Toronto, Toronto, Ontario, Canada; 2 Biostatistics Division, Princess Margaret Cancer Centre, Toronto, Ontario, Canada; 3 MD Program, Temerty Faculty of Medicine, University of Toronto, Toronto, Ontario, Canada; 4 Research Institute, Princess Margaret Cancer Centre, Toronto, Ontario, Canada; 5 Department of Radiation Oncology, Temerty Faculty of Medicine, University of Toronto, Toronto, Ontario, Canada; 6 Department of Medical Biophysics, Temerty Faculty of Medicine, University of Toronto, Toronto, Ontario, Canada; 7 Radiation Medicine Program, Princess Margaret Cancer Centre, Toronto, Ontario, Canada

## Abstract

**Importance:**

Secondary lymphedema is a common, harmful side effect of breast cancer treatment. Robust risk models that are externally validated are needed to facilitate clinical translation. A published risk model used 5 accessible clinical factors to predict the development of breast cancer–related lymphedema; this model included a patient’s mammographic breast density as a novel predictive factor.

**Objective:**

To investigate the external validity of a previously reported 5-factor model by applying it to an independent cohort of patients with breast cancer.

**Design, Setting, and Participants:**

This prognostic study collected data on a longitudinal cohort of patients with predominantly early-stage breast cancer treated with curative intent at the Princess Margaret Cancer Centre in Toronto, Canada between February 1, 2010, and July 31, 2014, with a median (IQR) follow-up of 4.3 (2.4-7.6) years. The 5 factors (age, body mass index, breast density, nodal burden, and use of axillary lymph node dissection [ALND]) were used as input into the established regression-based model. The analysis was performed from July 2 through August 29, 2024.

**Exposure:**

Lymphedema after breast cancer treatment.

**Main Outcomes and Measures:**

Lymphedema-free survival (LFS) was analyzed using Kaplan-Meier analysis, and sensitivity, specificity, and accuracy performance metrics of predicting breast cancer–related lymphedema were calculated.

**Results:**

A total of 101 female patients (median [IQR] age, 54.8 [48.8-62.3] years) were included in the analysis. These patients had localized or locoregional breast cancer treated with primary lumpectomy (90 [89%]) or mastectomy (11 [11%]); 75 (74%) had no axillary biopsy or sentinel lymph node biopsy; 26 (26%) had undergone ALND; and 38 (38%) had received chemotherapy, 101 (100%) received radiotherapy, and 64 (63%) received hormone therapy. Kaplan-Meier analysis showed a 2-year LFS of 97.5% (95% CI, 94.0%-100.0%) vs 65.0% (95% CI, 47.1%-89.7%) for the low- vs high-risk groups as defined by the 5-factor model (*P* < .001). The model sensitivity was 0.83 (95% CI, 0.52-0.98), specificity was 0.89 (95% CI, 0.80-0.94), and accuracy was 0.88 (95% CI, 0.80-0.94) for predicting breast cancer–related lymphedema.

**Conclusions and Relevance:**

These findings validate the performance of a 5-factor risk model for its prediction of 2-year LFS. Future clinical translation of this model can help with identifying patients at the highest risk of breast cancer–related lymphedema to facilitate closer surveillance and/or preventive management to improve health outcomes and quality of life.

## Key Points

**Question:**

Can a 5-factor risk model integrating age, body mass index, breast density, nodal burden, and use of axillary lymph node dissection accurately estimate 2-year lymphedema-free survival after cancer treatment in an independent cohort of patients with breast cancer?

**Findings:**

In this prognostic study of an independent cohort of 101 women with predominantly early-stage breast cancer, a multivariable 5-factor regression model performed well in predicting 2-year lymphedema-free survival.

**Meaning:**

These findings suggest that this externally validated 5-factor model has the potential to be a valuable tool for stratifying patients with breast cancer into low- and high-risk groups for breast cancer-related lymphedema.

## Teaser

This prognostic study investigates the external validity of a 5-factor model for estimating 2-year lymphedema-free survival after treatment for breast cancer.

## Introduction

Breast cancer consistently ranks among the top 3 most common cancers worldwide,^1^ and surgery, radiation therapy, and systemic therapy remain at the cornerstone of its management.^2^ Alongside these interventions, however, come potential long-term consequences, one of which is secondary lymphedema.^3^ Secondary lymphedema is characterized by the accumulation of lymphatic fluid in the extremities.^4^ It arises from damage to lymphatic vessels attributable to the cancer itself, surgery, radiation therapy, trauma, or infection.^4^ In developed nations, surgical lymph node dissection is its most common cause.^4^ From swelling to reduced mobility, from decreased body confidence to increased medical costs, the consequences of lymphedema are of physical, social, and economic concern.^5,6^

The occurrence of arm lymphedema is particularly high in patients with breast cancer because invasive breast cancer can metastasize to the lymph nodes of the axilla, thereby warranting surgical sampling and/or removal.^7^ Axillary lymph node dissection (ALND) is associated with a lymphedema incidence rate of 20%.^8^ Unfortunately, early manifestations of the disease are difficult to diagnose, and once under way, they worsen over time.^9^ Despite the existence of surgical and decongestive management techniques, lymphedema has no cure.^9^ Its posttreatment prevalence in patients with breast cancer combined with the widespread occurrence of breast cancer itself renders secondary lymphedema a pressing issue.

Early intervention based on risk assessments can reduce lymphedema progression and limit tissue damage.^10^ Risk models help predict the development of a disease based on relevant factors, enabling the early identification of high-risk individuals and facilitating the timely implementation of targeted monitoring and preventive measures.^10^ Existing models, however, often have poor accuracy when subjected to external validation outside the original dataset.^11^ For instance, the Cleveland Clinic Risk Calculator, an established risk model, had a predictive accuracy with an area under the curve of approximately 0.7 when initially developed^11^ but only 0.6 when externally tested.^12^ Furthermore, most lymphedema risk models are based on cancer- and treatment-specific factors, which on their own are insufficient to accurately determine risk.^13^ Patient-specific biological factors are needed to obtain a more comprehensive analysis and guide personalized treatment.^13^

A novel model developed by Kwan et al^13^ combines cancer and treatment data with patient characteristics. It uses multivariable linear regression to identify 5 key contributing factors that influence lymphedema occurrence and severity. These factors consist of 1 cancer factor (number of pathologic lymph nodes), 1 treatment factor (the performance of ALND), and 3 patient factors (age, body mass index [BMI], and mammographic breast density). The development of the model identified a new independent prognostic factor of mammographic breast density. Breast density is evaluated on mammograms and scored using the standardized Breast Imaging Reporting and Data System, where a lower score corresponds to lower density (ie, fattier breasts) and a higher score to higher density. Lower-density breasts were observed to be associated with a higher incidence of lymphedema. Using these 5 attributes, the model was able to predict the development of breast cancer–related lymphedema and its severity.

The model’s incorporation of patient-specific body composition characteristics poses benefits that extend beyond improved predictive accuracy. Unlike cancer- or treatment-related factors, patient-specific factors, such as body composition, may be more amenable to risk modification.^14^ Our aim was to investigate the external validity of the 5-factor model by applying it to an independent cohort of patients with localized breast cancer with long-term follow-up after cancer treatment. As most patients are diagnosed at a localized early stage of breast cancer, this is an important subpopulation to study.^15^ We hope to provide insight into the validity of this model and the generalizability of its application in modern clinical settings.

## Methods

### Study Design and Population

For this prognostic study, an independent study cohort was selected from a longitudinal cohort of patients with localized, nonmetastatic breast cancer who were prospectively recruited at the Princess Margaret Cancer Centre in Toronto, Ontario, Canada, from February 1, 2010, to July 31, 2014, for the purpose of evaluating the association between inflammatory biomarkers with fatigue symptoms. This cohort and its eligibility criteria have been previously described in detail by Shi et al.^16^ Specifically, for this validation study, exclusion criteria were inclusion in model development^13^ and missing clinical data. Research ethics board approval was obtained from the University Health Network with a waiver of informed consent as the study was a secondary analysis of this prospectively assembled cohort of patients with breast cancer. The Transparent Reporting of a Multivariable Prediction Model for Individual Prognosis or Diagnosis (TRIPOD) reporting guideline was followed.^17^

### Data Collection

Baseline data on patient demographics, diagnosis, and treatment details were retrieved for this study. Data collection included the 5 factors for the clinical risk model (age, BMI [calculated as weight in kilograms divided by height in meters squared], mammographic breast density, number of pathologic lymph nodes, and use of ALND) and long-term lymphedema outcomes. Data on race and ethnicity were not collected based on the original study design. Age was calculated as the number of years from the date of birth to the date of cancer surgery. Mammographic breast density was measured by licensed radiologists, classified based on the Breast Imaging Reporting and Data System scoring scale, and reported as a number from 1 to 4 based on the prior study.^13^ A standardized protocol was used for lymphedema severity measurements, which were recorded by occupational, physical, and/or registered massage therapists. For patients diagnosed with lymphedema, increased volume of the affected limb compared with the contralateral limb was confirmed using circumferential limb measurements at 7 standard anatomic points per arm based on prior methodology.^13^

### Statistical Analysis

The data analysis was performed between July 2 and August 29, 2024. Descriptive analysis of the study cohort included patient-, cancer-, and treatment-related characteristics. For each study participant, their 5 factors were used as input into the established model. This model equation is as follows^13^:

Lymphedema volume = −329 + [4 × Age] + [10 × BMI] – [37 × Mammographic Breast Density] + [13 × No. of Pathological Lymph Nodes] + [99 × ALND Treatment Use].

The high- and low-risk scores were defined as per the prior study as an equation score of greater than 200 and less than or equal to 200, respectively.^13^ We evaluated the performance of the model in predicting the occurrence of 2-year lymphedema-free survival (LFS). Kaplan-Meier event-free probability graphs were constructed from the initial surgical cancer treatment date until the occurrence of lymphedema or the last follow-up appointment. Events were demarcated by occurrence of lymphedema. High- and low-risk groups were defined as described previously by applying the model equation. Cox proportional hazard ratios (HRs) were calculated with reference to the low-risk group. Performance metrics (sensitivity, specificity, accuracy, positive predictive value, and negative predictive value) are reported. Sensitivity is defined as the probability of a positive test in an affected person and was calculated as the number of true positives divided by the sum of the true positives and false negatives.^18^ Specificity is defined as the probability of a negative test in an unaffected person and was calculated as the number of true negatives divided by the sum of the true negatives and false positives.^18^ Accuracy was calculated as the quotient of the sum of the true positives and true negatives divided by the total number of predictions.^19^ A negative predictive value indicates how likely it is for someone to be healthy in the case of a negative test result and was calculated as the number of true negatives divided by the sum of the true and false negatives.^20^ Precision (positive predictive value) was the number of true positives divided by the sum of the true and false positives.^20^ Based on the literature, the a priori target benchmarks were HR of 1.25,^21^ sensitivity of at least 0.80,^18^ specificity of at least 0.80, and accuracy of at least 0.85.^22^ The statistical significance threshold for all analyses was defined as *P* < .05 (2-sided). The statistical analyses were performed using R, version 4.3.1 (R Foundation) and SAS, version 9.4 (SAS Institute Inc).

## Results

**Figure 1.**
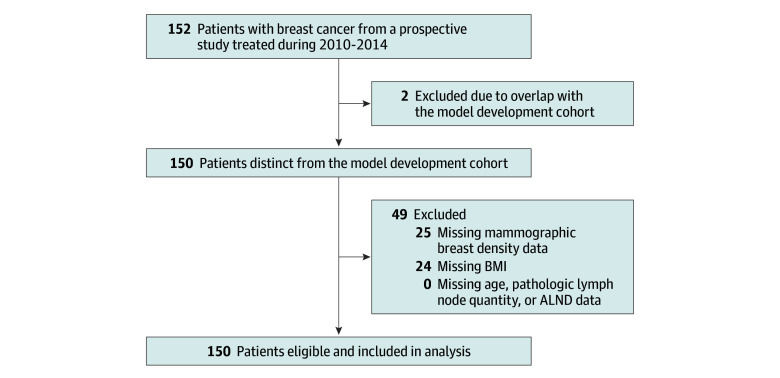
Patient Eligibility Selection of study participants included in the analysis. ALND indicates axillary lymph node dissection; BMI, body mass index.

 Among a total of 152 patients in the longitudinal cohort of patients with breast cancer, we excluded 2 who were previously included in the model development cohort in the prior publication at this institution.^13^ The remaining 150 patients were deemed eligible if they had available data on the 5 factors in the clinical risk model. From these 150 patients, 24 were excluded due to missing BMI data, and an additional 25 were excluded due to missing mammographic breast density data, resulting in 101 female participants (median [IQR] age, 54.8 [48.8-62.3] years; mean [SD] BMI, 26.6 [5.0]) included in the current analysis (Figure 1). These patients had localized or locoregional breast cancer treated with primary lumpectomy (90 [89%]) or mastectomy (11 [11%]); 75 (74%) had no axillary biopsy or sentinel lymph node biopsy; 26 (26%) underwent ALND; 38 (38%) received chemotherapy, 101 (100%) received radiation therapy, and 64 (63%) received hormone therapy. The distribution of baseline patient, cancer, and treatment characteristics are presented in the Table. The median follow-up time was 4.3 years (IQR, 2.4-7.6 years). Within the follow-up period, the overall incidence rate of lymphedema was 11.9% (12 of 101 patients). None of the patients developed severe lymphedema. The median time to lymphedema onset was 0.8 years (IQR, 0.7-1.3 years).

**Table.** Baseline Characteristics of Study Population (N = 101)

Characteristic	No. of participants (%)
Age, y	
Mean (SD)	55.7 (10.2)
Median (IQR)	54.8 (48.8-62.3)
Range	33.9-76.8
BMI	
Mean (SD)	26.6 (5.0)
Median (IQR)	26.0 (22.7-29.5)
Range	17.7-41.9
Mammographic breast density BI-RADS	
Almost entirely fatty	6 (6)
Scattered areas of fibroglandular density	46 (46)
Heterogeneously dense	42 (42)
Extremely dense	7 (7)
T category	
Tis	20 (20)
T1	52 (51)
T2	24 (24)
T3	5 (5)
N stage	
Nx	12 (12)
N0	60 (59)
N1	24 (24)
N2	3 (3)
N3	2 (2)
M stage	
M0	101 (100)
No. of pathologic nodes	
Mean (SD)	0.9 (2.6)
Median (IQR)	0 (0-1)
Range	0-19
No. of nodes removed	
Mean (SD)	6.1 (7.9)
Median (IQR)	2 (1-9)
Range	0-35
Primary surgery	
Lumpectomy	90 (89)
Mastectomy	11 (11)
Axillary surgery	
None	19 (19)
SLNB	56 (55)
ALND	26 (26)
Chemotherapy	
None	63 (62)
Neoadjuvant	9 (9)
Adjuvant	29 (29)
Hormone therapy	
No	37 (37)
Yes	64 (63)
Radiation therapy volume and fractionation	
Local breast radiotherapy, hypofractionated	67 (66)
Local breast radiotherapy, conventionally fractionated	9 (9)
Locoregional breast and lymph node radiotherapy, conventionally fractionated	25 (25)
Radiotherapy boost	
No boost	54 (53)
Boost	47 (47)
Lymphedema presence	
Yes	12 (12)
No	89 (88)
Follow-up time, y	
Mean (SD)	5.0 (3.3)
Median (IQR)	4.3 (2.4-7.6)
Range	0.1-11.8

Abbreviations: ALND, axillary lymph node dissection; BMI, body mass index (calculated as weight in kilograms divided by height in meters squared); BI-RADS, Breast Imaging Reporting and Data System; SLNB, sentinel lymph node biopsy.

**Figure 2.**
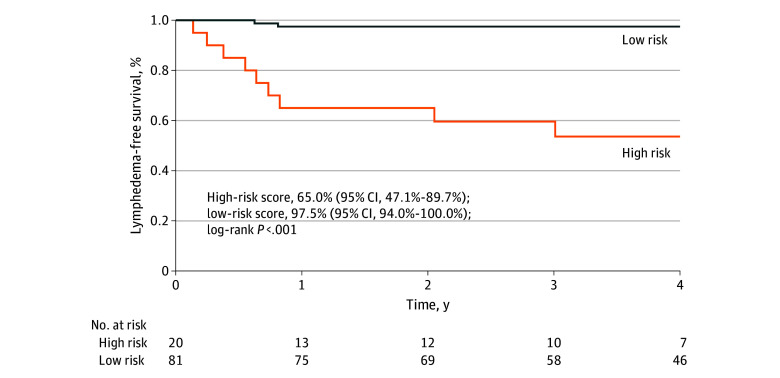
Prediction of 2-Year Lymphedema-Free Survival

Figure 2 depicts the LFS over time for patients with low- vs high-risk scores as predicted by the 5-factor clinical risk model. The 2-year LFS for the low-risk group was 97.5% (95% CI, 94.0%-100.0%), whereas that of the high-risk group was 65.0% (95% CI, 47.1%-89.7%) (*P* < .001). The corresponding HR was 22.24 (95% CI, 4.80-103.09; *P* < .001), as calculated using Cox proportional hazards regression with reference to the low-risk group. This HR exceeds the target benchmark of 1.25,^21^ suggesting a clinically significant difference between groups.

Furthermore, the model performance was evaluated in terms of sensitivity, specificity, accuracy, positive predictive value, and negative predictive value. Sensitivity was 0.83 (95% CI, 0.52-0.98), specificity was 0.89 (95% CI, 0.80-0.94), accuracy was 0.88 (95% CI, 0.80-0.94), positive predictive value was 0.50 (95% CI, 0.27-0.73), and negative predictive value was 0.98 (95% CI, 0.91-1.00). The performance of the 5-factor model exceeded the target benchmarks of 0.80 for specificity and sensitivity and 0.85 for accuracy.

## Discussion

With the high prevalence of breast cancer and the common occurrence of secondary lymphedema as a side effect of its treatment, there is a need for accurate and robust tools to facilitate early identification of patients with breast cancer at risk of lymphedema. In this prognostic study, a comprehensive 5-factor lymphedema risk model^13^ incorporating patient-, cancer-, and treatment-specific characteristics was identified to be accurate for prediction of 2-year LFS and was shown to be robust through successful validation in an independent cohort of patients with breast cancer.

Most of the baseline characteristics of the cohort used in this external validation study were comparable to those of the population from which the training and internal validation cohorts of the original study were drawn^13^ and are consistent with the characteristics of the general breast cancer population. For instance, the median age was 52.3 years (IQR, 45.9-60.1 years) in the previous study population and 54.8 years (IQR, 48.8-62.3 years) in ours. Likewise, the mean (SD) BMI was 26.9 (6.0) in the training cohort, 27.8 (5.9) in the internal validation cohort, and 26.6 (5.0) in this external validation cohort. Breast density distributions were also similar. However, this cohort differed from the original cohorts in terms of the number of pathologic lymph nodes and the proportion of patients who had undergone ALND. In terms of pathologic lymph nodes, the mean (SD) number was 2.3 (4.2) and 2.8 (6.3) for the initial training and internal validation cohorts, respectively, but only 0.9 (2.6) for our study, showing the ability to extrapolate the established model to patients with an earlier stage of breast cancer. As for ALND, the fraction of patients who had undergone ALND in the original study was approximately double that of this cohort, thereby showing the utility of this model in the modern era in which ALND is more sparingly used.^23^

A key reason for externally validating models is that when models are created through training and internal validation, patients are selected in the same way and from the same time period; hence, the model is likely to perform similarly when applied to both groups. External validation cohorts offer a new set of participants selected through other methods and therefore provide insight into the generalizability of the model. With a sensitivity, specificity, and accuracy of 0.83, 0.89, and 0.88, respectively, we found that the model performs well at predicting lymphedema in an external cohort, suggesting good generalizability.

All 5 risk factors in this model are supported by previous literature. The integration of patient-, cancer-, and treatment-related factors contributes to the performance of this model in a complementary fashion. Older age is associated with diseases of the vasculature, which can affect lymphatic flow and contribute to lymphedema.^24^ BMI has been widely reported to be an important risk factor for lymphedema, with higher BMI corresponding to higher risk.^25,26,27^ Nodal burden and the level of ALND are associated with the extent of damage to the lymphatics and have been shown in multiple studies to be positively correlated with lymphedema risk.^8,28^ When it comes to age, BMI, and ALND, other risk models, such as the Cleveland Clinic Risk Calculator, have incorporated these variables in their prognostication as well,^12^ which is a testament to their prognostic value. However, the clinical use of ALND is declining.^23^ In the future, the contribution of this individual risk factor may have decreased value, while use of other patient- and cancer-related risk factors may have increased importance.

This study’s 5-factor model includes a newer risk factor of mammographic breast density, which was introduced in the prior study^13^ and serves as an alternative and local measure of adiposity that complements BMI. The previous study reported that patients with high breast adiposity may have an increased likelihood of lymphedema development. High adiposity has been associated with metabolic syndrome, which comprises a cluster of metabolic abnormalities that manifest as abnormal adiposity, increased triglyceride levels, increased blood pressure, and decreased high-density lipoprotein cholesterol.^29^ Metabolic syndrome can lead to chronic inflammation and impaired lymphatic growth.^30^ Additionally, while BMI focuses on overall adiposity, mammographic breast density focuses on tissue composition closer to where breast and lymph node surgery occurs, rendering it a potentially more relevant local factor for predicting breast cancer-related lymphedema in the upper extremities.

New clinical practice guidelines have highlighted the importance of early intervention and preventive management for breast cancer–related lymphedema.^31^ For patients receiving ALND, this risk model could be used to predict their risk of lymphedema before implementing preventive management in order to avoid overtreatment. On the other hand, for patients with limited axillary surgery, this model could be used for accurate risk stratification. By using routine clinical data to stratify patients into a low vs high risk of lymphedema, the model offers a useful tool to identify at-risk patients who may need targeted surveillance and prophylactic strategies in the future.

### Strengths and Limitations

This study has both strengths and limitations. Strengths include the use of a high-quality clinical dataset derived from a cohort of patients with breast cancer recruited at one of the largest cancer centers in North America, where lymphedema outcomes were uniformly assessed by trained clinic personnel and reported using a standard dictation template. The follow-up time was sufficiently long for the outcome. This patient population, although recruited from the same institution as the model development cohort, had slightly different cancer and treatment characteristics (eg, lower pathologic lymph node burden, decreased use of ALND), which provided a new cohort of patients to assess the robustness and generalizability of the model. We acknowledge limitations in the incidence of lymphedema in this cohort that is representative of predominantly patients with early-stage breast cancer, which may contribute to variation in the HR and to the low positive predictive value. The relatively small sample size of this study is also a potential limitation; however, between this and the previously published study,^13^ this model has now been applied to 3 different clinical cohorts: a development cohort, internal validation cohort, and external validation cohort. Due to the limited number of patients with severe lymphedema in our study, we were unable to validate the original model’s prediction of severe lymphedema, which is an uncommon condition.

Future research directions include validation of this 5-factor model at more institutions, which can also facilitate its clinical translation and adoption at other centers. We acknowledge that the practice of lymphedema monitoring and management is highly variable within and between countries. To obtain high-quality clinical datasets with lymphedema outcomes to facilitate validation, the practice of measuring lymphedema severity needs to be adopted by other institutions as standard practice. For institutions that use newer technologies to refine lymphedema detection, such as bioimpedance spectroscopy, further evaluation is required to determine the role of this 5-factor model in predicting subclinical lymphedema. Finally, continued reassessment of the optimal risk factors for breast cancer–related lymphedema may be required as the treatment of breast cancer advances.

## Conclusions

This prognostic study of a longitudinal cohort of patients with predominantly early-stage breast cancer externally validated the performance of a 5-factor lymphedema risk model for its prediction of 2-year LFS. The factors incorporated into this model are all readily accessible by clinicians, and the successful performance of the model on an external cohort of patients with breast cancer is a positive indicator of its generalizability.

## Notes

Supplement.
Data Sharing Statement
