# Supplementary material for: External Validation of a 5-Factor Risk Model for Breast Cancer–Related Lymphedema
Source: JAMA Netw Open. 2025 Jan 21;8(1):e2455383. doi: 10.1001/jamanetworkopen.2024.55383 (PMC11751742; doi:10.1001/jamanetworkopen.2024.55383)
Supplement: Supplement. — Data Sharing Statement [file jamanetwopen-e2455383-s001.pdf]

Data Sharing Statement

Lin. External Validation of a 5-Factor Risk Model for Breast Cancer–Related Lymphedema. *JAMA Netw Open*. Published January 21, 2025. doi:10.1001/jamanetworkopen.2024.55383

Data
Data available: No

Additional Information
Explanation for why data not available: Anonymized data may be shared with other researchers on request by contacting the corresponding author. Requesters will be required to sign a data sharing agreement.
